# Towards Aptamer-Targeted Drug Delivery to Brain Tumors: The Synthesis of Ramified Conjugates of an EGFR-Specific Aptamer with MMAE on a Cathepsin B-Cleavable Linker

**DOI:** 10.3390/pharmaceutics16111434

**Published:** 2024-11-11

**Authors:** Vladimir A. Brylev, Ekaterina V. Ryabukhina, Ekaterina V. Nazarova, Nadezhda S. Samoylenkova, Evgeny L. Gulyak, Ksenia A. Sapozhnikova, Fatima M. Dzarieva, Alexey V. Ustinov, Igor N. Pronin, Dmitry Y. Usachev, Alexey M. Kopylov, Andrey V. Golovin, Galina V. Pavlova, Dmitry Yu. Ryazantsev, Vladimir A. Korshun

**Affiliations:** 1 Shemyakin-Ovchinnikov Institute of Bioorganic Chemistry, Miklukho-Maklaya 16/10, 117997 Moscow, Russiaevgeny.gulyak@gmail.com (E.L.G.); ksapozh@mail.ru (K.A.S.); v-korshun@yandex.ru (V.A.K.); 2 Burdenko National Medical Research Center of Neurosurgery, 4th Tverskaya-Yamskaya 16, 125047 Moscow, Russia; 3 Lumiprobe RUS Ltd., Kotsyubinskogo 4, bld. 3, 121351 Moscow, Russia; 4 Institute of Higher Nervous Activity and Neurophysiology, Butlerova 5A, 117485 Moscow, Russia; 5 Department of Chemistry, Lomonosov Moscow State University, Leninskie Gory 1-3, 119991 Moscow, Russia; kopylov.alex@gmail.com (A.M.K.);; 6 Department of Microbiology, Virology and Immunology, Sechenov First Moscow State Medical University, Trubetskaya 8, 119991 Moscow, Russia; 7 Department of Medical Genetics, Sechenov First Moscow State Medical University, Trubetskaya 8, 119991 Moscow, Russia

**Keywords:** aptamers, branched conjugates, drug delivery, cytostatic payload, brain tumors

## Abstract

**Background/Objectives:** Targeted delivery of chemotherapeutic agents is a well-established approach to cancer therapy. Antibody–drug conjugates (ADCs) typically carry toxic payloads attached to a tumor-associated antigen-targeting IgG antibody via an enzyme-cleavable linker that releases the drug inside the cell. Aptamers are a promising alternative to antibodies in terms of antigen targeting; however, their polynucleotide nature and smaller size result in a completely different PK/PD profile compared to an IgG. This may prove advantageous: owing to their lower molecular weight, aptamer-drug conjugates may achieve better penetration of solid tumors compared to ADCs. **Methods:** On the way to therapeutic aptamer–drug conjugates, we aimed to develop a versatile and modular approach for the assembly of aptamer–enzymatically cleavable payload conjugates of various drug–aptamer ratios. We chose the epidermal growth factor receptor (EGFR), a transmembrane protein often overexpressed in brain tumors, as the target antigen. We used the 46 mer EGFR-targeting DNA sequence GR-20, monomethylauristatin E (MMAE) on the cathepsin-cleavable ValCit-*p*-aminobenzylcarbamate linker as the payload, and pentaerythritol-based tetraazide as the branching point for the straightforward synthesis of aptamer–drug conjugates by means of a stepwise Cu-catalyzed azide–alkyne cycloaddition (CuAAC) click reaction. **Results:** Branched aptamer conjugates of 1:3, 2:2, and 3:1 stoichiometry were synthesized and showed higher cytotoxic activity compared to a 1:1 conjugate, particularly on several glioma cell lines. **Conclusions:** This approach is convenient and potentially applicable to any aptamer sequence, as well as other payloads and cleavable linkers, thus paving the way for future development of aptamer–drug therapeutics by easily providing a range of branched conjugates for in vitro and in vivo testing.

## 1. Introduction

More than 80% of diagnosed malignant primary brain tumors are gliomas [1]. Gliomas are formed from glial cells of the brain, such as astrocytes, oligodendrocytes, ependymal cells, and others [2]. According to the World Health Organization, the malignancy of gliomas is classified into grades 1–4 [3]. Grades 3–4 are the most common and the most malignant: they are anaplastic, and poorly differentiated, with a median survival rate of less than 3 years for grade 3 and less than 15 months for grade 4 [4]. This group includes IDH-mutant anaplastic astrocytoma (grade 3) and glioblastoma (GBM) (grade 4) [2]. GBM is one of the most malignant tumors—it always recurs even after intensive treatment. It originates from astrocytes, and what causes it and how to prevent it is largely unknown. Typical survival after the diagnosis is 15 months, with less than 10% of people surviving more than 5 years with combined treatment [5]. There are several complicating factors that make glioblastoma difficult to treat: GBM cells often are resistant to conventional therapies, but the brain is sensitive to therapy and has a poor capacity for recovery. An additional reason is that many drugs are unable to cross the blood–brain barrier (BBB) in order to have an effect on the tumor; however, high-grade glioblastoma often renders the BBB permeable [6].

Targeted drug delivery using specific antigens expressed on the surface of the tumor cells was an obvious idea for the treatment of glioblastoma [7]. GBM is characterized by intense expression of the epidermal growth factor receptor (EGFR or HER1) on the membrane, including mutant tumor-specific forms, such as EGFRvIII and others [8]. EGFR is a transmembrane receptor protein that has a population of protein ligands with extracellular localization, and it is essential for the life cycle of normal cells. Several mutations of EGFR can lead to its overexpression with a constant activation of the receptor and uncontrolled cell proliferation. That is why EGFR is a highly popular target in drug discovery [9], with a number of approved antibody-based therapeutics [10,11,12,13,14,15]. In particular, EGFR-targeted antibody–drug conjugates (ADCs) for brain tumor therapy are under extensive development [16,17,18,19,20,21].

In general, ADCs are an excellent example of targeted payload delivery to tumors; their efficacy, pharmacodynamics, and pharmacokinetics have been extensively studied [22,23,24,25]. The concept of targeted drug delivery is based on a highly tumor-specific interaction between the targeting molecule and the tumor marker, which consists of binding and internalization into the cell; the subsequent release of the toxic payload results in the death of the tumor cell [26]. The last two decades have been crucial for the development of targeted cancer therapy; however, the choice of payloads [26,27,28] and cleavable linkers is still rather limited [24]. Moreover, the difficulty of site-specific antibody modification raises the issue of ADC homogeneity and control of the drug-to-antibody ratio [29,30]. There are several examples of ADCs being developed for brain tumor therapy [31,32,33,34]; however, the unique biology of brain tumors requires specifically tailored drug development strategies [35]. Although it has been observed that the BBB is permeable in many cases of glioblastoma [6], the passage of ADCs across the BBB is still an issue that requires investigation [36].

Aptamers are oligonucleotide sequences capable of recognizing a specific target with an affinity comparable to that of an antibody. Their therapeutic potential is well recognized [37,38,39,40], including anticancer applications [41,42,43,44,45]. Several aptamers (E07, CL4, mE07, R9, TuTu22, U2) have been developed for targeting EGFR [46,47,48,49,50,51].

Inspired by the active development of selective antibody-based anticancer drugs, we aimed to target EGFR using aptamers loaded with the anticancer drug monomethyl auristatin E (MMAE), which is an inhibitor of tubulin polymerization. Potential advantages of aptamer–drug conjugates are (1) high affinity to a specific target and (2) the possibility of site-specific aptamer modification (modern oligonucleotide synthesis allows control of all modifications and homogeneity). However, there are some shortcomings associated with low stability in the bloodstream because nucleic acids are not adapted to function under such conditions. Even so, the problem of plasma stability has been overcome for antisense oligonucleotides using carbohydrate and phosphate modifications [52].

**Figure 1:**
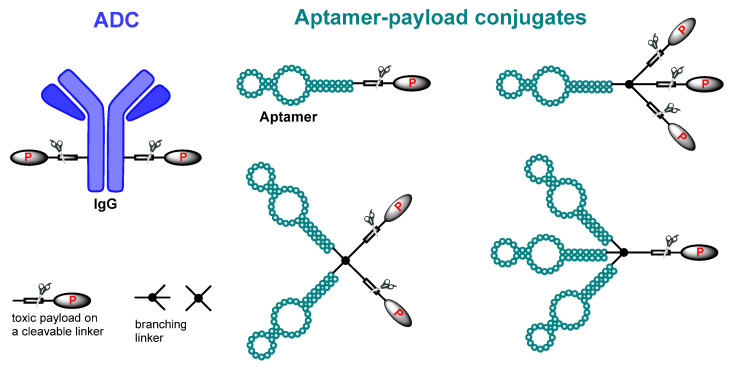
Sketched structures of common antibody–drug conjugates (drug-to-antibody ratio may vary from 1 to 8) and aptamer–drug conjugates prepared in this study.

In this paradigm, the aptamer serves as the delivery vehicle, but the efficiency of internalization of the aptamer-receptor complex remains unclear. As such, we set out to synthesize conjugates of different architectures, including multivalent constructs that may be capable of inducing EGFR clustering, which has been shown to promote internalization [53]. In general, multimerization has been found to be a useful approach to improving the specificity and affinity of aptamers [54,55,56,57,58,59,60,61]. Thus, our goal was to develop a methodology of aptamer–payload conjugation through a cathepsin B-cleavable linker and the synthesis of multivalent branched conjugates containing two and three aptamer moieties (Figure 1). Cathepsin B-cleavable peptide linkers have been conjugated to oligonucleotides before, but with rather sophisticated approaches [62,63,64]. Here, we propose a simple and reliable synthetic procedure for such conjugates.

## 2. Materials and Methods

### 2.1. General Methods

HPLC was carried out on an Agilent 1100 instrument (Santa Clara, CA, USA) using a xBridge column, inner diameter 4.6 mm, length 250 mm, (Waters, Milford, MA, USA) and linear gradient from 5 to 85% MeCN in a gradient of triethylammonium acetate from 0.15 M to 0.02 M for 40 min. Protected 2′-deoxyribonucleoside 3′-phosphoramidites were purchased from Hongene Biotech (Shanghai, PRC), and Unylinker-CPG (1000 Å) was purchased from ChemGenes (Wilmington, MA, USA); 5′-alkyne phosphoramidite was purchased from Lumiprobe (Hunt Valley, MD, USA). The alkyne derivative of the payload, **Alkyne-Val-Cit-PABC-MMAE** [65], as well as diazide (1,11-diazido-3,6,9-trioxaundecane) [66] and tetraazide (6,6-bis(5-azido-2-oxapentyl)-1,11-diazido-4,8-dioxaundecane) [67], was prepared as described.

### 2.2. Oligonucleotide Synthesis

5′-Alkyne-modified oligonucleotides were assembled in an ASM-2000 DNA synthesizer (Biosset, Novosibirsk, Russia) using the standard phosphoramidite method on a universal support and with an alkyne reagent [68]. Sequences:

[Alkyne]-GGTCGCTTATCTGCACTCGGA (**A1**),
[Alkyne]ACGCACCATTTGTTTAATATGTTTTTTAATTCCCCTTGTGGTGTGT (**GR20**).

The oligonucleotides were treated with 10% diethylamine in MeCN for 10 min, cleaved from the support, and deprotected using AMA—1:1 (*v*/*v*) conc. aq. ammonia and 40% aq. Methylamine—for 30 min at 65 °C. The target oligonucleotides were purified using preparative HPLC. Purity was controlled by denaturing 12% PAGE and HPLC.

Oligonucleotides labeled with AF488 were prepared by the CuAAC click reaction of alkyne oligonucleotides with AF488 azide (Lumiprobe, Hunt Valley, MD, USA) using a procedure similar to that described below (Section 2.3).

### 2.3. General Method for Click Reaction of Oligonucleotides with Polyazides and MMAE Alkyne, One-Pot Procedure

Alkyne-modified oligonucleotides (30 nmol) in 2 mL tubes were charged with pre-degassed deionized water (60 μL), DMSO (60 μL), ascorbic acid in a 2M TEAA buffer (15 μL), and an azide solution in DMSO (5 μL of 100 mM for excess of di- or tetraazide or 15 μL of 1 mM for lack of tetraazide). The obtained mixture was thoroughly stirred. A 10 mM solution of CuSO_4_∙5H_2_O—TBTA premix in 55% aq. DMSO was added (6 μL) to the reaction mixtures. Tightly closed tubes were kept in darkness at an ambient temperature for 3 h. The reaction mixtures were precipitated with 2% LiClO_4_ in acetone (1.5 mL) at −20 °C for 30 min. Centrifugation at 10,000 rcf produced the precipitate, which was rinsed with pure acetone, dried, and dissolved in deionized water (60 μL). The crude azido-modified oligonucleotides reacted with a 2-molar excess, according to total azido-groups of alkyne-modified MMAE, in the same manner as described above.

### 2.4. General Method for Conjugate Purification

The reaction mixture after the final click reaction with alkyne-modified MMAE was precipitated with 2% LiClO_4_ in acetone (1.5 mL) at −20 °C for 30 min. Centrifugation at 10,000 rcf produced the precipitate, which was rinsed with pure acetone, dried, and dissolved in 50% aq. formamide (100 μL). An analysis of reaction mixtures was performed by denaturing 10% polyacrylamide gel electrophoresis (length 10 cm, 8 M urea, 1× TBE buffer), carried out for 30 min at a constant voltage of 350 V. The bands on gels were visualized by shadows on precoated aluminum F_254_ TLC plates with a fluorescent indicator (Merck Millipore, Darmstadt, Germany) using UV light with λ = 254 nm.

The products were separated by denaturing 10% or 15% polyacrylamide gel electrophoresis (depending on size). The bands containing products of click reactions were carefully cut out with a scalpel. The obtained slices were frozen at −20 °C, crushed, and eluted twice with deionized water (1000 μL total). The eluates were desalted using NAP-10 gel columns according to the standard protocol. The purity of MMAE-modified oligonucleotides was determined using HPLC, analytical denaturing 14% PAGE, and electrospray mass-spectrometry.

### 2.5. Cell Lines

Human colon cancer HCT-116, human epidermoid carcinoma A-431, human glioblastoma U-251 and U-87, and human lymphoblast K-562 cells were cultured in an RPMI 1640 medium supplemented with 10% fetal bovine serum (FBS), 2 mM L-glutamine, 100 U/mL penicillin, and 100 µg/mL streptomycin (all—PanEco, Moscow, Russia) at 37 °C and 5% CO_2_. 

### 2.6. Flow Cytometry

The adhesion cells (HCT-116, A-431, U-251, U-87) were detached from the culture flasks by a 0.02% EDTA solution. The staining of cells (10^5^ cells) with an AF488-labeled EGFR-specific GR20 aptamer conjugate or control AF488-labeled oligonucleotide A1 conjugate (1000 nM, 100 nM, 10 nM, 1 nM) was performed for 1 h at 37 °C and 5% CO_2_. After incubation, the cells were washed twice with a DPBS buffer (PBS with calcium and magnesium salts, PanEco, Moscow, Russia). The samples were then immediately analyzed using a LongCyte C2060 flow cytometer (Challenbio, Beijing, PRC). In each sample, at least 50 000 events were collected. The relative fluorescence intensity (RFI) of EGFR expression in each cell line was calculated as the ratio of specific fluorescence of cell staining with the AF488-labeled aptamer conjugate and autofluorescence of control unstained cells. The data were analyzed using FlowJo v10.9 software.

### 2.7. Cell Viability Assay

Cell viability was analyzed using the colorimetric MTT method with 3-(4,5-dimethylthiazol-2-yl)-2,5-diphenyltetrazolium bromide (PanEco, Moscow, Russia). Serial 5-fold dilutions of aptamer and control oligonucleotide conjugates from 1000 nM to 0.0128 nM were made in a 100 µL RPMI medium and were placed into the wells of 96-well plates. Then, cells were added into the wells (5 × 10^3^ cells per well) and incubated for 72 h at 37°C with a 5% CO_2_ atmosphere. Then, the MTT solution (250 μg/mL) was added to each sample and the cells were incubated for 3h. Optical density (OD) was registered at 540 nm using a Hidex Sense Beta Plus microplate reader (Hidex, Turku, Finland). Cell viability was calculated as

Cell viability = 100% × (OD_treated cells_ − OD_blank_)/(OD_control cells_ − OD_blank_)

## 3. Results

### 3.1. Synthesis of Conjugates

The DNA aptamer GR20 [69] was selected for EGFR targeting, and the highly cytotoxic monomethyl auristatin E (MMAE) was used as the payload. To connect the two, the clinically validated cathepsin-cleavable Val-Cit-PABC moiety was chosen as a linker that effects payload release upon exposure to proteolytic enzymes in the lysosome [70,71]. The key conjugation reaction was Cu(I) azide–alkyne cycloaddition (CuAAC), a rapid and convenient transformation that is (bio)orthogonal with respect to the aptamers, MMAE, and the Val-Cit-PABC linker.

**Scheme 1:**
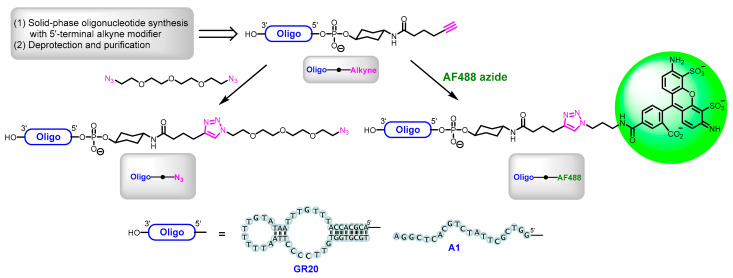
Synthesis of 5′-azido- and 5′-AF488-modified oligonucleotides.

Synthetic oligonucleotides (**GR20** and the unstructured control **A1**) were prepared using the standard protocol by 5′-alkyne modification [68]. Then, CuAAC was carried out with an excess of diazide (1,11-diazido-3,6,9-trioxaundecane) or AF488 azide in the liquid phase [66], yielding azido-modified or AF 488-labeled oligonucleotides, respectively (Scheme 1).

**Scheme 2:**
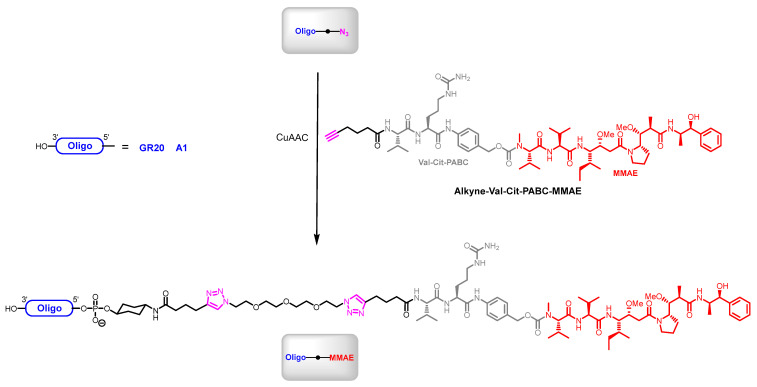
Synthesis of 1:1 oligonucleotide-5′-payload conjugates; red—MMAE payload, gray—cathepsin-cleavable linker.

The 5′-azide-modified oligonucleotides were further coupled with an alkyne derivative of the payload already containing the enzymatically cleavable linker **Alkyne-Val-Cit-PABC-MMAE** [65] (Scheme 2). The CuAAC click reaction was performed in an aqueous solution and produced high yields of oligo–payload conjugates with a 1:1 stoichiometry (Scheme 2).

**Scheme 3:**
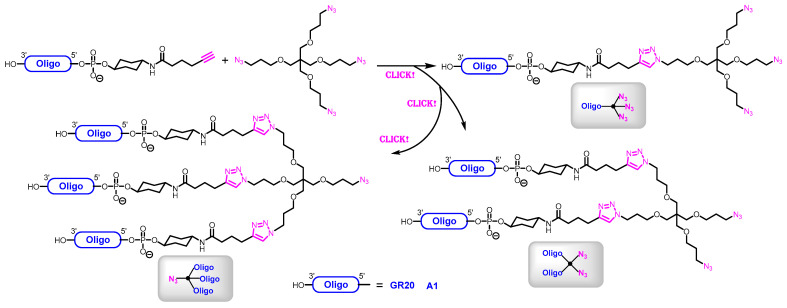
CuAAC modification of 5′-alkyne-oligonucleotides with tetraazide.

The click reaction of alkyne oligonucleotides with tetraazide (6,6-bis(5-azido-2-oxapentyl)-1,11-diazido-4,8-dioxaundecane) yielded a series of branched conjugates—Oligo(N_3_)_3_, (Oligo)_2_(N_3_)_2_, and (Oligo)_3_N_3_. The stoichiometry of the resulting products was tuned by controlling the excess of the tetraazide to the alkyne oligonucleotide [67] (Scheme 3).

**Scheme 4:**
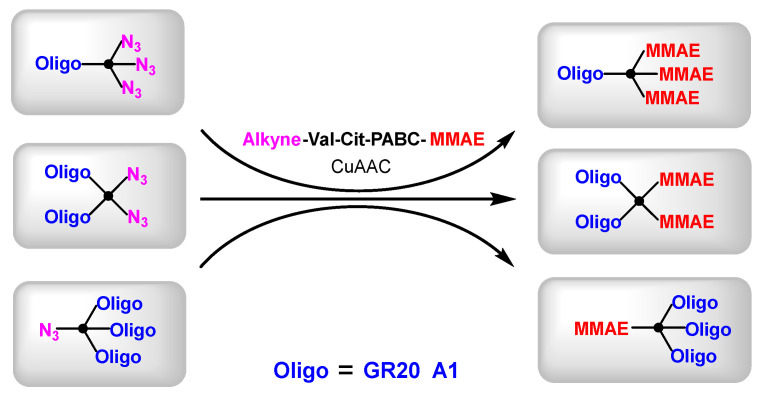
CuAAC modification of 5′-alkyne-oligonucleotides with tetraazide. For structures of the payload and the cleavable linker, see Scheme 2.

The azido-modified oligonucleotides were converted to payload conjugates via CuAAC with alkyne-modified Val-Cit-PABC-MMAE. The stoichiometry of the conjugates was either 1:3, 2:2, or 3:1 (Scheme 4). The target conjugates were purified using preparative HPLC and analyzed using denaturing PAGE and HPLC.

Thus, a series of MMAE-loaded conjugates of the GR20 aptamer and A1 control oligonucleotides with four different stoichiometries were prepared.

**Figure 2:**
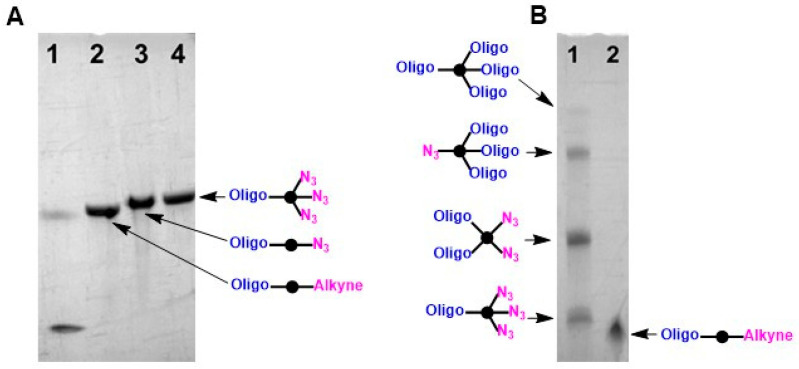
The 10% analytical denaturing PAGE. (**A**) 1—molecular weight markers bromophenol blue (low) and xylene cyanol (high), 2—alkyne-modified GR20, 3—the click reaction of alkyne–GR20 with 1,11-diazido-3,6,9-trioxaundecane, 4—the click reaction of alkyne–GR20 with an excess of tetraazide. (**B**) 1—the click reaction of alkyne–GR20 with tetraazide in a 3:1 molar ratio, 2—alkyne-modified GR20.

**Figure 3:**
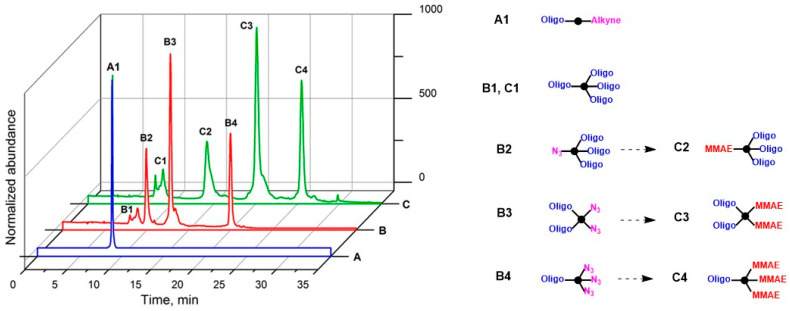
Typical HPLC profile of click reaction between alkyne–GR20 and tetraazide. A—HPLC profile of pure starting alkyne-modified aptamer GR20. B—HPLC profile of click reaction between tetraazide and alkyne-modified GR20. C—HPLC profile of click reaction of azido-modified GR20 mixture with alkyne-modified MMAE.

The click reaction with alkyne-modified MMAE was performed in a one-pot manner with PAGE and HPLC control of intermediate azide-modified conjugates of the GR20 aptamer (Figure 2 and Figure 3). The initial alkyne–GR20 (Figure 2) had the highest electrophoretic mobility. Electrophoretic control of the click reaction between alkyne–GR20 and an excess of diazide or tetraazide showed the formation of products, OligoN_3_ and Oligo(N_3_)_3_, respectively (Figure 2A). The click reaction with tetraazide produced a set of products with stoichiometries of 1:3, 2:2, and 3:1 (Figure 2B).

In the case of the click reaction with tetraazide, no unreacted alkyne–GR20 was observed by electrophoresis (Figure 2B). The B1 peak (Figure 3) was determined to be the peak of the tetra-clicked conjugate, so in a subsequent reaction with alkyne–MMAE it was not transformed into a payload conjugate and we observed it in its unchanged form as peak C1 (Figure 3). All azide-containing conjugates of the GR20 aptamer with the corresponding peaks B2, B3, and B4 were transformed into payload conjugates, and detected as peaks C2, C3, and C4, respectively (Figure 3). In a similar way, the MMAE conjugates of the unstructured control oligonucleotide A1 were synthesized.

### 3.2. Flow Cytometry and Cell Viability Assay

**Figure 4:**
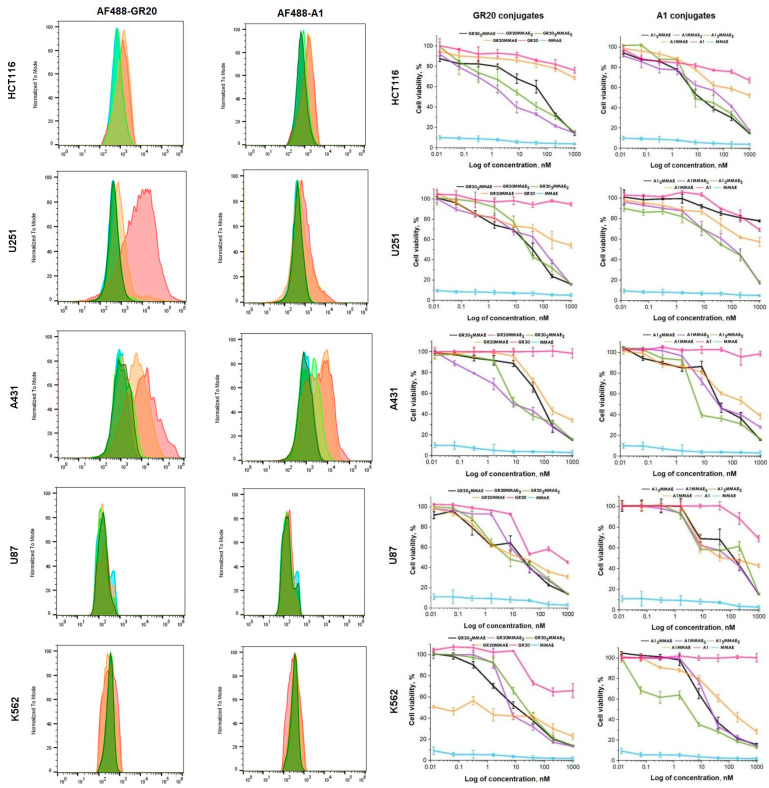
Flow cytometry (**left panel**) and MTT cell viability assay of conjugates (**right panel**) obtained in five cell lines—HCT116, U251, A431, U87, K562. (**Left panel**) blue—cell autofluorescence; AF488–oligonucleotide concentrations: red—1000 nM, orange—100 nM, green—10 nM, dark green—1 nM.

To perform the initial screening of binding specificity and to assess the biological effect, we relied on well-known data [72] on EGFR expression levels in several cancer cell lines. Five cell lines were used, including one non-EGFR-expressing one (K562), one overexpressing one (A431), and three with comparable expression levels (U87, U251, HCT116). Flow cytometry was performed on all five cell lines to assess specificity. A derivative of the GR20 aptamer with AF488 was used as a fluorescent probe for staining the cells. Oligonucleotide A1 modified with the same dye was used as a control. AF488 was chosen because of its permanent negative charge and high hydrophilicity, which potentially reduces non-specific interactions with cells. Flow cytometry shows (Figure 4, left panel) that, even at high concentrations, AF488-GR20 and AF488-A1 show no specific binding to EGFR-negative K562 cells. U251 cells showed high binding to the fluorescently labeled aptamer and very weak binding to the fluorescently labeled control non-aptamer oligonucleotide AF488-A1. The difference in fluorescence intensities was two orders of magnitude. Cells overexpressing EGFR were also successfully stained with the fluorescent derivative AF488-GR20, and the difference from the control AF488-A1 was one order of magnitude. U87 and HCT116 cells were stained weakly with the fluorescent aptamer AF488-GR20, and there was virtually no difference from the control AF488-A1. The optimal concentration for cell staining was 1000 nM. Lower concentrations did not result in a significant level of detectable fluorescence compared to unstained cells. Thus, a reliable preferential binding of GR20 compared to A1 was revealed in one expressing and one superexpressing cell line. Binding specificity of the fluorescent aptamer derivative is probably determined not only by the level of EGFR expression, but also by the features of its localization in the cell membrane. The biological properties of the four MMAE conjugates, as well as the GR20, MMAE controls, and A1 conjugates, were studied on the same cell lines. In all cases, the MMAE conjugates showed quite strong activity. The controls without MMAE did not show activity. The branched conjugates always showed better activity values compared to the linear conjugates. The activity of conjugates in a cell viability assay was in the range of IC_50_ 1–10 nM (Figure 4, right panel).

## 4. Discussion

The CuAAC click reaction is a reliable method for the preparation of oligonucleotide conjugates [73,74,75,76]. Alkyne derivatives of oligonucleotides are readily available. The MMAE payload is a short, chemically stable peptide with well-developed procedures for its modification with various functional enzymatically cleavable linkers. MMAE is a potent tubulin inhibitor that has been successfully used as a payload in several approved ADCs on a Val-Cit-PABC linker [77]. The 46 mer DNA sequence, the EGFR-targeting aptamer GR20, was used as a model oligonucleotide for the construction of conjugates. DNA aptamers are typically structured, forming one or more hairpins under normal conditions.

**Scheme 5:**
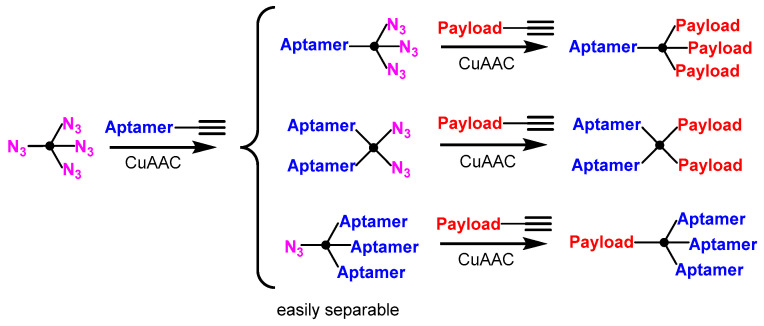
Two-step CuAAC assembly of various aptamer–payload conjugates.

The conjugates contain an oligonucleotide, a branching point, and linker/payload modules. These units are coupled together in two similar CuAAC click steps (Scheme 5). The first step is a reaction of a tetraazide with an alkyne-modified aptamer. It yields three products with aptamer/azide stoichiometries of 1:3, 2:2, and 3:1. The ratio of reaction products is controlled by the ratio of starting compounds. These azido intermediates containing one, two, or three oligonucleotide moieties are easily separable by PAGE or HPLC, and are therefore readily available as pure individual compounds. The latter can be easily converted into the corresponding aptamer–payload conjugates (Scheme 4).

The payload is introduced into the modification step as an alkyne-modified cleavable linker–based derivative. Almost all common enzymatically cleavable linkers and toxic payloads tolerate CuAAC conditions, thus making the approach versatile and suitable for the full range of available linkers and payloads, including potential new ones [78]. Moreover, this modular principle can be further developed in the future for the construction of conjugates carrying two different payloads or peptides to enhance transport across the BBB.

We found that the branched aptamer conjugates were more potent than their 1:1 analog. The combination of two payloads in one conjugate with different cellular targets is considered as a promising approach to overcome tumor resistance to therapy [30,78,79]. The development of peptides/proteins targeting the transferrin receptor is a hot topic for drug delivery to the brain [80,81,82]. Therefore, we will continue to develop synthetic approaches for conjugates containing recognizing, transport-enhancing, and therapeutic moieties.

## 5. Conclusions

In summary, we have developed a general approach to the synthesis of oligonucleotide–MMAE conjugates of various stoichiometries with an enzymatically cleavable linker. The method is based on a two-step click approach. In the first step, alkyne-modified oligonucleotides are readily converted to azido derivatives via a click reaction with di- or tetraazide in solution. The resulting azido-modified oligonucleotides/aptamers are then reacted with an alkyne-modified MMAE derivative containing an enzyme-cleavable linker. Branched and linear conjugates of the EGFR-targeting aptamer GR20 and a model unstructured oligonucleotide were synthesized and tested on various cancer cell lines. The general modular approach is potentially applicable to the assembly of conjugates of any oligonucleotide sequences, payloads, and cleavable linkers with a desired stoichiometry.

## Data Availability

All data will be made available by the corresponding author upon reasonable request.
